# The beneficial metabolic actions of prolactin

**DOI:** 10.3389/fendo.2022.1001703

**Published:** 2022-09-23

**Authors:** Yazmín Macotela, Xarubet Ruiz-Herrera, Dina I. Vázquez-Carrillo, Gabriela Ramírez-Hernandez, Gonzalo Martínez de la Escalera, Carmen Clapp

**Affiliations:** Instituto de Neurobiología, Universidad Nacional Autónoma de México (UNAM), Querétaro, Mexico

**Keywords:** prolactin levels, homeoFIT-PRL, metabolically healthy and unhealthy obesity, metabolic homeostasis, insulin resistance, homeorhetic response

## Abstract

The role of prolactin (PRL) favoring metabolic homeostasis is supported by multiple preclinical and clinical studies. PRL levels are key to explaining the direction of its actions. In contrast with the negative outcomes associated with very high (>100 μg/L) and very low (<7 μg/L) PRL levels, moderately high PRL levels, both within but also above the classically considered physiological range are beneficial for metabolism and have been defined as HomeoFIT-PRL. In animal models, HomeoFIT-PRL levels counteract insulin resistance, glucose intolerance, adipose tissue hypertrophy and fatty liver; and in humans associate with reduced prevalence of insulin resistance, fatty liver, glucose intolerance, metabolic syndrome, reduced adipocyte hypertrophy, and protection from type 2 diabetes development. The beneficial actions of PRL can be explained by its positive effects on main metabolic organs including the pancreas, liver, adipose tissue, and hypothalamus. Here, we briefly review work supporting PRL as a promoter of metabolic homeostasis in rodents and humans, the PRL levels associated with metabolic protection, and the proposed mechanisms involved. Finally, we discuss the possibility of using drugs elevating PRL for the treatment of metabolic diseases.

## Introduction

Defining the role of prolactin (PRL) in metabolism has been challenging due to contrasting findings demonstrating positive and negative effects of PRL on metabolic homeostasis. This contradiction is disentangled after realizing that PRL levels and the physio-pathological context influence the direction of PRL action (1). Low and very high PRL levels are deleterious to the metabolism, whereas medium and moderately high levels are usually beneficial.

PRL action is necessary to maintain metabolic homeostasis, as the absence or reduction of PRL signaling due to the lack of PRL receptors (PRLR) or low PRL levels associate with exacerbated metabolic alterations, particularly in the context of a metabolic challenge or disease. In humans, low PRL levels associate with increased prevalence of metabolic diseases (1). In contrast, patients with overweight and obesity (OW/OB) having elevated PRL levels show better metabolic profiles than BMI-matched patients with lower PRL values (2–6), to imply that elevated PRL is a mechanism dealing with metabolic challenge.

The mechanisms by which PRL promotes metabolic homeostasis involves actions in different metabolic organs. A detailed description of the levels of PRL and their cellular and molecular mechanisms mediating metabolic benefits warrant further research. Also, a careful evaluation of drugs that elevate PRL levels is needed in the context of metabolic diseases.

## Prolactin promotes metabolic homeostasis in rodents

Serum PRL decreases in rodents with obesity, diabetes, and insulin resistance (2, 7–10), suggesting a role for reduced PRL levels in the pathophysiology of metabolic diseases. As a proof of concept, PRL treatment in mice and rats with streptozotocin (STZ)-induced diabetes or diet-induced obesity improves their metabolic profile (2, 11, 12), whereas PRLR null mice with STZ-induced diabetes or diet-induced obesity show a more severe disease phenotype (2, 13). Moreover, mice lacking PRLR in the liver become insulin resistant, whereas insulin resistant obese mice (db/db mice lacking leptin receptors) overexpressing the PRLR in the liver show improved insulin sensitivity (14).

In addition, PRL action is required to deal with the metabolic challenges of pregnancy, a state characterized by hyperphagia, excessive adiposity, and physiological insulin resistance to redirect nutrients towards the fetus (15–17). Pregnant mice null for the PRLR in the pancreas, specifically in β-cells, develop gestational diabetes (18–20), due to deficient pancreatic β-cell hyperplasia and hyperinsulinemia (21).

Moreover, PRL reduces metabolic alterations in lactating pups nursed by dams consuming a high fat diet (HFD) during lactation. The obesogenic milk from HFD-fed dams has 50% less PRL compared to the milk from dams fed a chow diet (22). Pups consuming the obesogenic-hypoprolactinemic milk develop obesity, excessive adiposity, severe insulin resistance, and fatty liver at weaning; whereas when their HFD-fed mothers or themselves receive exogenous PRL during lactation, metabolic alterations are ameliorated (22). These findings support PRL in maternal milk exerting beneficial metabolic effects in lactating pups, and low PRL levels in milk contributing to the maternal obesogenic diet-induced metabolic disease in pups.

## Elevated prolactin levels as a mechanism to counteract metabolic alterations in humans

Low PRL levels associate with a higher prevalence of type 2 diabetes (T2D), insulin resistance, glucose intolerance, metabolic syndrome (MS), adipose tissue (AT) dysfunction, β-cell dysfunction, non-alcoholic fat liver disease (NAFLD), and cardiovascular events, whereas moderately high PRL levels correlate with metabolic protection in all these instances (**Table 1**).

**Table 1:** Moderately high PRL serum levels associate with lower incidence of metabolic disease.

Metabolic disease	Population	PRL level associated with lower disease incidence or prevalence (μg/L)
**T2D**	Women Women & men Pregnancy Women w/GDM	>15.8 (23), 18.4 (24) >12.9 (25), >11.5 (26), Q4 (27, 28) >115 Lower postpartum risk (29) >78.7 postpartum, lower risk of future T2D (30)
**Insulin resistance**	Men Women & men Children Women w/PCOS Women & men w/obesity	≥12.0 (2) ≥12.0 (3), >11.5 (26) Inverse association with PRL levels (31) 7.9 (32) >14.9 (33), Inverse association with PRL levels (34) Inverse association with PRL levels (5)
**Fasting glucose levels & HbA1c**	Women w/T1D Women & men w/obesity Women & men	Inverse association with PRL levels (35) 19.2 (6) 30.5 (4), >11.5 (26), >12.9 (25), Q4 (28) Inverse association with PRL levels (31)
**MS**	Children Men w/SD Women w/PCOS	7.9 (32) >11.1-35 (36), Inverse association with PRL levels (37) >7.0 (38)
**Adipose tissue dysfunction**	Women & men Men Women w/PCOS Women & men w/obesity	≥12.0 (3) ≥12.0 (2) Inverse association with PRL levels (34) 19.2 (5, 6)
**Metabolically unhealthy obesity**	Women & men w/obesity Women & men	19.2 (5, 6) 30.5 (4)
**Beta cell dysfunction**	Pregnancy Women w/PCOS	>115 Lower postpartum risk (29) >14.9 (33)
**Dyslipidemia**	Women & men Women & men w/obesity Women w/PCOS	30.5 (4) Inverse association with PRL levels (5) >7.0 (38), >15.9 (39)
**Major CVE**	Men w/SD	> 12 – 35 (40)
**NAFLD**	Women & men	>12.8 (41)

Clinical studies within the last 12 years showing an inverse association between PRL circulating levels and risk, prevalence or incidence of metabolic diseases. Abbreviations: Q, quartile; T2D, type 2 diabetes; GDM, gestational diabetes mellitus; PCOS, polycystic ovary syndrome; T1D, type 1 diabetes; HbA1c, glycosylated hemoglobin; MS, metabolic syndrome; SD, sexual dysfunction; CVE, cardiovascular event; NAFLD, non-alcoholic fatty liver disease.

Moderately high PRL levels (16–35 μg/L) associate with lower prevalence of T2D and even predict a reduced incidence of T2D 10 years later (23). PRL levels in the 4^th^ quartile correlate with lower incidence (23, 25, 29) or prevalence (24, 26–28, 30, 42) of T2D (**Table 1**), and PRL levels are inversely related to fasting glucose levels and glycosylated hemoglobin (HbA1c) values (4, 25, 26, 28, 31, 35, 36) in both men and women. Consistently, high serum PRL in pregnancy predicts a lower risk of postpartum prediabetes/diabetes (29), and in women with gestational diabetes mellitus, lower PRL levels at 6 to 9 weeks postpartum associate with a higher future risk of developing T2D in a 10-year follow up (30) (**Table 1**). T2D and other metabolic alterations derive from insulin resistance, i.e., the inability of insulin to activate a normal insulin response on its target cells. Moderately elevated PRL levels associate with increased insulin sensitivity in men (2, 3, 5, 26, 31), women (3, 5, 26, 31, 33, 34) and even children (32) (**Table 1**).

Insulin resistance can derive from AT dysfunction and occur in parallel to β-cell dysfunction. High PRL levels associate with reduced AT dysfunction and predict smaller adipocytes (reduced hypertrophy) in visceral AT (2, 3, 5, 6, 34), the type of fat that, in excess, associates with metabolic alterations and disease severity (43–46). Regarding β-cell function, pregnant women with high PRL levels have a lower postpartum risk of developing diabetes and β-cell dysfunction (29), and women with polycystic ovary syndrome (PCOS) with PRL levels in the 4^th^ quartile show lower prevalence of β-cell dysfunction (33) (**Table 1**).

The MS represents a group of alterations that elevate the risk of cardiovascular disease, stroke, and T2D, and consists of high blood pressure, hyperglycemia, abdominal obesity, and abnormal cholesterol and triglyceride levels (47). Moderately high PRL levels associate with lower prevalence of MS in children (32) and in adult patients suffering from certain conditions, such PCOS in women (38), and sexual dysfunction (SD) in men (36, 37). Also, high PRL levels in men with SD are associated with protection from major cardiovascular events (40). However, in the general adult population a correlation between PRL and MS has not been found (3, 25). When only dyslipidemia is evaluated, an inverse association occurs between PRL levels and total cholesterol, LDL cholesterol, and triglyceride levels (4, 5, 38, 39).

Another parameter closely linked to metabolic disease is a proinflammatory environment. In subjects with obesity, moderately high PRL levels associate with lower levels of interleukin 6 in children (32) and tumor necrosis factor-α (TNF-α) in adults (4).

**Figure 1:**
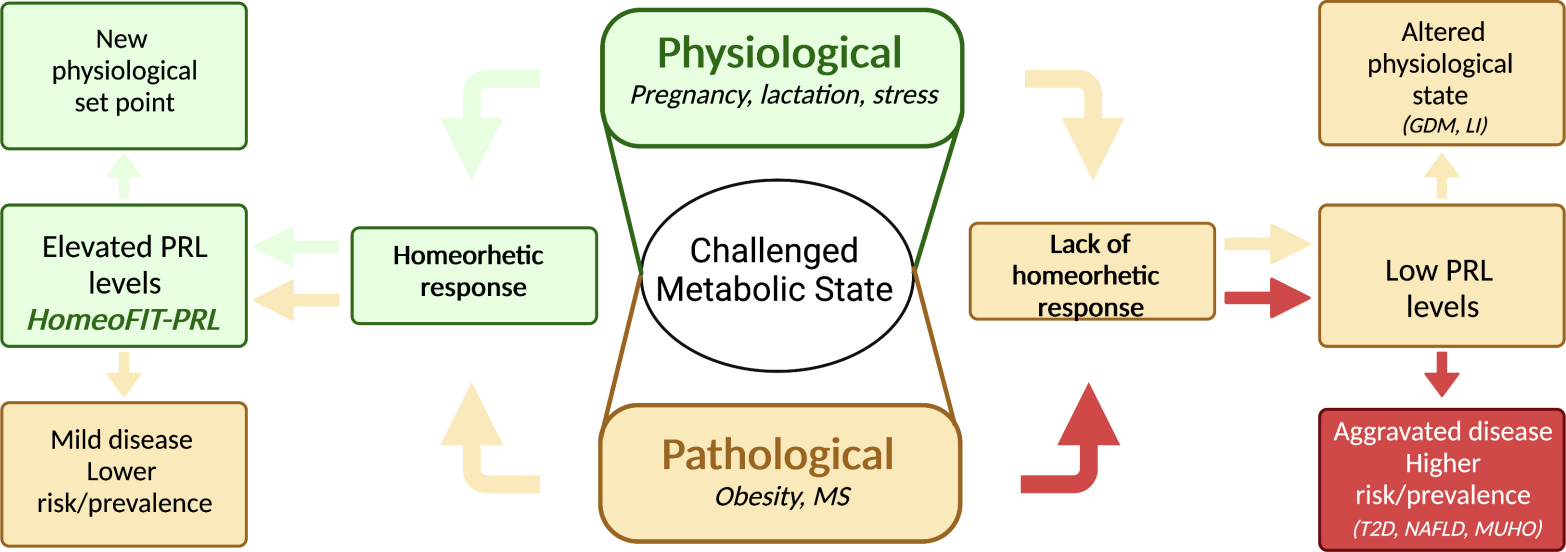
Elevated prolactin levels are part of a homeorhetic response upon metabolic challenges. A challenged metabolic state can be either physiological or pathological; in both cases a homeorhetic response includes elevated prolactin (PRL) levels, allowing a series of metabolic adaptations to deal with the physio-pathological demand. In a physiological challenge, such as pregnancy, lactation, or stress, this response leads to a new physiological set point (green arrows), whereas in a pathological challenge, such as obesity, it leads to a milder disease or protection from disease risk (yellow arrows, left side of figure). If the homeorhetic response fails, PRL levels do not rise and remain low instead, leading to altered physiological states (i.e., gestational diabetes mellitus, GDM, lactation insufficiency, LI, anxiety) (yellow arrows, right side of figure), or to aggravated disease with higher disease risk or prevalence (red arrows, right side of figure). MS, metabolic syndrome, T2D, type 2 diabetes, NAFLD, non-alcoholic fatty liver disease; MUHO, metabolically unhealthy obesity. Created in BioRender.com.

Most studies in humans show that moderately high PRL levels are not associated with obesity itself, the exception being a study in children (32). This observation can be explained by the fact that some subjects with obesity remain metabolically healthy (metabolically healthy obesity - MHO), or at least show fewer metabolic alterations. Indeed, subjects having MHO have increased circulating PRL levels as compared to those with metabolically unhealthy obesity (MUHO) (4–6). Moreover, logistic regression analysis showed PRL as an independent predictor of MHO (6). Patients with obesity and high PRL (HP) levels displayed reduced blood glucose, total and LDL cholesterol, triglyceride, and TNFα levels than patients with obesity and normal PRL (NP) levels. Also, after sleeve gastrectomy, patients in the HP group showed reduced PRL levels, whereas those in the NP group have increased PRL levels (4). Similarly, patients with OW/OB with higher PRL levels had a better metabolic profile than those with lower PRL values. Interestingly, PRL levels decreased once metabolic parameters improved following bariatric surgery (5) (**Table 1**). These studies support that increased PRL levels are protective against metabolic diseases and return to basal values after the metabolic challenge is resolved (**Figure 1**).

Another metabolic disease associated with low PRL levels is NAFLD. Patients with NAFLD show lower PRL levels than control subjects and those with severe hepatic steatosis have even lower PRL values than patients with a mild to moderate disease (41) (**Table 1**). Moreover, PRL levels are part of a mathematical model to diagnose the presence and severity of NAFLD (48).

The association between low PRL levels and higher prevalence of metabolic diseases also stands for postmenopausal women and middle-aged and elderly men (23, 36), implying its independence from gonadal status. Because PRL levels may decrease with aging, it remains to be determined whether the HomeoFIT-PRL range differs between young vs. middle-age or elderly individuals.

## The right prolactin levels for metabolic maintenance and protection – *not too much and not too little*

While low and very high PRL levels have deleterious metabolic consequences, a specific range of PRL values is beneficial for metabolism. This PRL range includes levels in the normal physiological range (7 to 25 μg/L) but also levels above (25 to 100 μg/L). The latter, previously claimed as hyperprolactinemia, have been defined as HomeoFIT-PRL (Homeostatic Functionally Increased Transient Prolactinemia) (1), since they occur in response to physiological or pathological challenges and respond to it by favoring metabolic homeostasis (**Figure 1**).

In healthy individuals PRL levels are usually within the classical normal range <25 μg/L. However, some physiological challenges elevate PRL in a transient manner, such as intense exercise, acute stress, sleep, and sexual arousal (49). These conditions together with reproductive states (pregnancy and lactation) can be categorized as conditions that trigger a homeorhetic response, meaning the orchestrated or coordinated control of body metabolic tissues necessary to maintain a physiological state (defined by Bauman and Currie) (50). Moreover, the association between moderately elevated PRL levels and a beneficial metabolic phenotype supports elevated PRL levels in obesity as part of a homeorhetic response occurring both, under physiological and pathological challenges (**Figure 1**).

Altogether, PRL levels ranging from 7 to100 μg/L are beneficial for metabolism. PRL values are in the lower end of this range under healthy physiological conditions (outside reproductive states); however, in the context of a metabolic challenge they are likely to increase towards maintaining metabolic homeostasis and return to basal when the stressor/challenge is eliminated. Conversely, patients experiencing a metabolic challenge, such as obesity, that are unable to respond by increasing PRL levels, are more prone to suffer from metabolic alterations than those upregulating their PRL levels (**Figure 1**).

Elevated PRL levels derived from prolactinomas are not part of a response to a metabolic challenge, they result from a diseased state (tumor) and are not considered HomeoFIT-PRL (and are usually above 100 μg/L). It is expected that normalization of PRL levels in subjects with prolactinomas associate with a healthier metabolic profile, if the PRL levels achieved by the treatment remain in the healthy range (>7μg/L).

## Mechanisms mediating the beneficial metabolic action of prolactin

PRL actions favoring metabolism are the result of its pleiotropic action reflected by the presence of the PRLR in almost every tissue in the body, including the main metabolic organs —pancreas, liver, adipose tissue, muscle, intestine, and hypothalamus— where beneficial metabolic actions and mechanisms of PRL have been described (51, 52).

### Pancreatic β-cells

**Figure 2:**
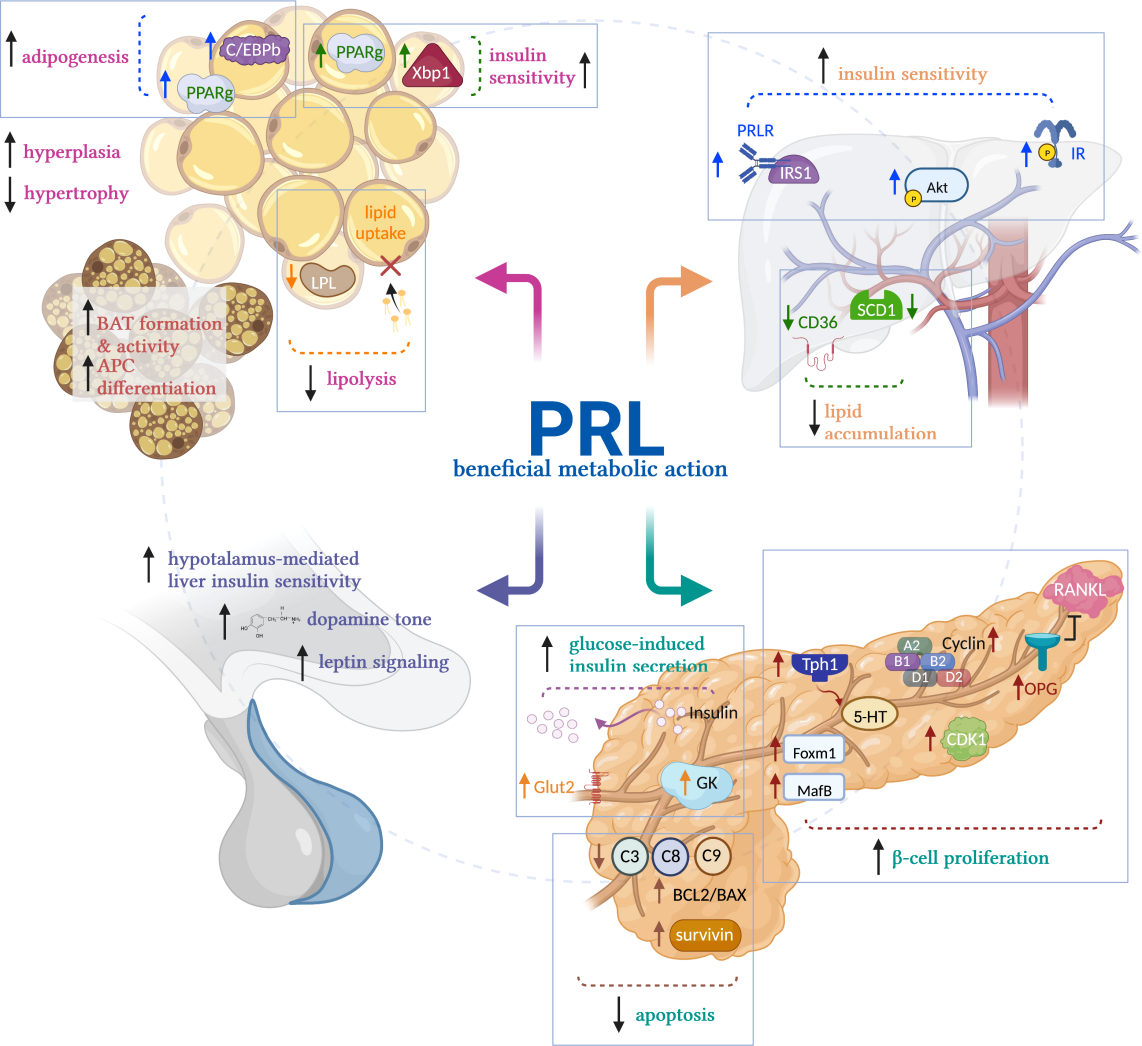
Mechanisms of prolactin’s beneficial metabolic actions. Prolactin (PRL) promotes metabolic homeostasis acting on the main metabolic tissues. In white adipose tissue, PRL reduces adipocyte size by stimulating lipolysis and reducing LPL activity, preventing lipid uptake. Also, it stimulates insulin sensitivity by activating PPARg and Xbp1s and promotes adipogenesis by activating CEBP/b and PPARg, favoring the healthy expansion of adipose tissue by hyperplasia vs hypertrophy in obesity conditions. In brown adipose tissue (BAT), PRL promotes adipocyte differentiation and BAT formation and activity in newborns. In liver, PRL promotes insulin sensitivity by its canonical signaling STAT5, and by activation of IRS1 and AKT. PRL also reduces liver lipid accumulation by reducing the activity of SCD1 and CD36, preventing aggravated fatty liver in NAFLD. In pancreas, PRL promotes β-cell proliferation, inhibits their apoptosis, and elicits glucose-induced insulin secretion. In hypothalamus, PRL promotes dopamine release and stimulates leptin signaling, inducing hypothalamus-mediated liver insulin sensitivity. LPL, lipoprotein lipase; PPARg; peroxisome proliferator-activated receptor-g; Xbp1s, spliced form of X-box-binding protein-1; CEBP/b, CCAAT/enhancer-binding protein beta; PRLR, prolactin receptor; IR, insulin receptor; IRS1, insulin receptor substrate 1; AKT, Protein kinase B; SCD1, stearoyl-CoA desaturase 1; CD36, fatty acid translocase; Tph1, tryptophan hydroxylase 1; 5-HT, serotonin; OPG, osteoprotegerin; RANKL, receptor activator of NF-kB ligand; Foxm1, forkhead box M1; MafB, MAF BZIP transcription factor B. Created in BioRender.com.

PRL stimulates the proliferation and survival of β-cells (53, 54), promotes glucose-induced insulin secretion (53), stimulates pancreas development during the perinatal stage (55), and is essential for β-cell expansion during pregnancy (18, 19, 56). The mechanisms that mediate PRL effects on β-cells involve increased osteoprotegerin synthesis, leading to the inhibition of receptor activator of NF-kB ligand pathway, an inhibitor of β-cell proliferation (57); increased survivin levels (58), elevated expression of the transcription factors Foxm1 and MafB, increased cyclin activity, and higher islet serotonin production *via* Tph1 synthesis, all promoting β-cell proliferation (18, 56). Also, PRL leads to the inhibition of extrinsic and intrinsic apoptosis pathways (54) and improved glucose sensitivity through increased glucokinase and glucose transporter 2 expression (19, 59, 60) (**Figure 2**).

### Liver

PRL regulates liver growth (61) and liver metabolic function. Increased PRLR expression in liver stimulates both liver and systemic insulin sensitivity, whereas reduced hepatic PRLR expression results in tissue and whole-body insulin resistance (14). Also, PRL reduces hepatic lipid accumulation by inhibition of the expression of the fatty acid transporter CD36 and the lipid synthesis enzyme, SCD1 (41, 62). Consistently, there is an inverse association between PRL levels and hepatic CD36 expression, and the PRLR decreases in the liver of patients with NAFLD (41). Thus, PRL prevents fatty liver disease. Mechanistically, the activation of STAT5 downstream of the PRLR mediates the insulin sensitizing effects of PRL (14). PRLR interacts with IRS1 (63) and promotes the phosphorylation of AKT (64), two key members of the insulin signaling pathway. Upregulating the hepatic PRLR in combination with systemic insulin treatment enhances the phosphorylation of the insulin receptor and of AKT in mouse liver, whereas reducing the expression of the PRLR by adenovirus-shRNA impairs insulin-induced liver phosphorylation of IR and AKT (14) (**Figure 2**). Moreover, the PRLR is regulated by the level of hepatic insulin resistance/sensitivity, i.e., it is downregulated in insulin resistant conditions and upregulated in insulin sensitive states (14).

### Adipose tissue

PRL acts on the AT to regulate lipid metabolism and promote adipogenesis and healthy AT expansion (65). PRL inhibits lipid uptake *via* reduced lipoprotein lipase activity in human fat (66) and inhibits lipolysis in rat and human AT (67). PRL contributes to adipocyte differentiation in the adipocyte cell lines NIH-3T3 and 3T3-L1, by stimulating the activation of STAT5, and of the adipogenic transcription factors C/EBPb and PPARg (68, 69). PRL is essential for brown fat formation and activity in newborn mice, and for brown preadipocyte differentiation (70). The PRLR is present in AT from rodents and humans and PRL is secreted by human AT (65, 66, 71), while obesity decreases PRL release from human fat (67). In PRLR null mice, there is either decreased or no change in fat mass (2, 72–74) depending on age, fat depot, and genetic background. C57BL/6 PRLR null mice fed an HFD, show increased adiposity and exacerbated adipocyte hypertrophy in AT (2). In obese rats, PRL treatment stimulates the healthy expansion of AT by promoting adipocyte hyperplasia and reducing visceral adipocyte hypertrophy, *via* increased expression of transcription factors PPARg and Xbp1s, both favoring adipogenesis and insulin sensitivity (2) (**Figure 2**).

### Hypothalamus

PRL promotes insulin sensitivity, at least in part, by central actions on the hypothalamus. Increased PRLR expression in the hypothalamus stimulates whole body insulin sensitivity, whereas reduced PRLR expression results in insulin resistance and glucose intolerance (75). PRL effects on the hypothalamus lead to vagal signals that promote increased liver insulin sensitivity (75). Also, in 90% pancreatectomized rats, intracerebroventricular infusion of PRL increases liver insulin sensitivity, inhibits β-cell apoptosis, and reduces body weight and adiposity by increasing hypothalamic dopamine levels and leptin signaling (76) (**Figure 2**).

## Prolactin elevating drugs in the treatment of metabolic diseases

Several drugs elevate PRL circulating levels, mainly those that act as dopamine D2 receptor blockers, including first- and second-generation antipsychotics and medications treating gastrointestinal symptoms, antidepressants, antihypertensives, and others (77, 78). The use of antipsychotics has been associated to the development of metabolic alterations; however, a recent meta-analysis, evaluating the metabolic actions of 18 antipsychotics in around 26,000 patients with schizophrenia (79), showed a large variation in the metabolic side-effects of antipsychotics. Some drugs had clear adverse effects increasing body weight, triglyceride levels, cholesterol levels, and glucose levels (olanzapine, clozapine, and quetiapine), while others showed neutral or even positive metabolic outcomes, with very mild or no effects on body weight and triglyceride levels, and some reducing LDL cholesterol and glucose levels (aripiprazole, brexpiprazole, cariprazine, lurasidone, ziprasidone and amisulpiride). Regarding the effect of these drugs on PRL levels (77), some of the drugs exerting beneficial metabolic actions present a moderate to high risk for elevating PRL levels (77), whereas the drugs causing adverse metabolic actions have minimal to moderate risk for elevating PRL levels (77). This and other studies (80, 81) support those metabolic adverse effects derived from treatment with antipsychotic drugs not being associated with elevated PRL levels. Attention on drugs that exert beneficial metabolic effects by elevating PRL to HomeoFIT-PRL levels with negligible adverse actions is warranted.

One example is amisulpiride, a D2/D3 antagonist shown to reduce glucose levels in humans (79) and in diet-induced obese mice (82). The proposed beneficial metabolic action of amisulpiride at low doses involves increasing dopaminergic activity by preferentially blocking presynaptic D2/D3 receptors (83). Also, amisulpiride seems to stimulate insulin secretion by pancreatic β-cells (82). Therefore, given the positive metabolic effects of amisulpiride at low doses and its capacity to increase PRL levels, it is worth testing whether this and other benzamides can improve metabolic outcomes in obesity conditions.

Another benzamide, levosulpiride, is being tested in a clinical trial on patients with diabetic retinopathy and diabetic macular edema to elevate PRL levels and favor its conversion into vasoinhibin, the antiangiogenic, anti-vasopermeability PRL-derived fragment (84). The results of this clinical study raise the possibility to explore the potential therapeutic benefits of levosulpiride on obesity-derived metabolic alterations.

The fact that bromocriptine quick release (Cycloset), a PRL-lowering drug, is an FDA-approved treatment for T2D questions the association between low PRL levels and high prevalence of T2D. This controversy can be explained by the fact that dopamine and PRL act through different mechanisms to promote metabolic homeostasis. There is a morning surge of dopaminergic activity in the central nervous system that lowers insulin resistance and hyperglycemia, and this surge is reduced in patients with T2D (85). Accordingly, by counteracting such reduction, treatment with bromocriptine benefits glucose homeostasis. Also, bromocriptine increases glucose tolerance in diet-induced obese mice that are PRL deficient (86). Whether normalizing PRL levels in bromocriptine-treated patients leads to further metabolic improvements is unclear and needs to be investigated.

## Conclusions and future perspectives

PRL is present in the circulation throughout life and, particularly in humans, its levels are comparable between sexes, highlighting the role of PRL in physiology beyond reproduction. PRL senses the metabolic status of an individual, and upon physiological and pathological metabolic challenges its levels rise as part of an homeorhetic response, allowing organisms to adequately adjust to such demands. On the other hand, the inability to elevate PRL levels in challenged conditions aggravates metabolic diseases and alters physiological outcomes.

Key questions remain to be addressed such as: *1*) What are the signals that increase PRL levels in metabolically healthy individuals and what prevents such elevations in metabolically unhealthy individuals? *2*) Does the pharmacological elevation of PRL levels in metabolically unhealthy individuals improve their health outcomes? *3*) Are changes in PRL (either decreased or elevated levels) in metabolic diseases part of a larger cascade of altered responses? and, if so, what is the upstream or leading regulator of the cascade? *4*) What and how is the PRLR regulated in different physio-pathological conditions and a tissue-specific manner?

Future studies should focus on answering these questions, evaluating the benefit of PRLR-specific agonists, and carefully testing whether the current D2 receptor antagonists at low doses may be useful in the treatment of metabolic diseases due to their PRL-elevating properties. Understanding the underpinnings of PRL actions on metabolism in physiological and pathological conditions will help target this hormone to improve health outcomes.

## Author contributions

YM wrote manuscript. XR-H prepared figures. XR-H, DV-C, GR-H, GE and CC reviewed, edited, and approved manuscript. All authors contributed to the article and approved the submitted version.

## Funding

This work was supported by grants from CONACYT 284771, and UNAM DGAPA-PAPIIT IN207321 to YM.

## Acknowledgments

We thank Jessica Gonzalez Norris for critically editing the manuscript.

## Conflict of interest

The authors declare that the review was conducted in the absence of any commercial or financial relationships that could be construed as a potential conflict of interest.

## Publisher’s note

All claims expressed in this article are solely those of the authors and do not necessarily represent those of their affiliated organizations, or those of the publisher, the editors and the reviewers. Any product that may be evaluated in this article, or claim that may be made by its manufacturer, is not guaranteed or endorsed by the publisher.

## References

[1] Macotela Y Triebel J Clapp C. Time for a new perspective on prolactin in metabolism. Trends Endocrinol Metab (2020) 31(4):276–86. doi: 10.1016/j.tem.2020.01.004 32044206

[2] Ruiz-Herrera X de Los Rios EA Diaz JM Lerma-Alvarado RM Martinez de la Escalera L Lopez-Barrera F. Prolactin promotes adipose tissue fitness and insulin sensitivity in obese males. Endocrinology (2017) 158(1):56–68. doi: 10.1210/en.2016-1444 27805870

[3] Ponce AJ Galvan-Salas T Lerma-Alvarado RM Ruiz-Herrera X Hernandez-Cortes T Valencia-Jimenez R. Low prolactin levels are associated with visceral adipocyte hypertrophy and insulin resistance in humans. Endocrine (2020) 67(2):331–43. doi: 10.1007/s12020-019-02170-x 31919769

[4] Wang X Ma B Li G Sheng C Yang P Gao J. Glucose-lipid metabolism in obesity with elevated prolactin levels and alteration of prolactin levels after laparoscopic sleeve gastrectomy. Obes Surg (2020) 30(10):4004–13. doi: 10.1007/s11695-020-04771-2 32700179

[5] Liu J Wang Q Zhang L Fu J An Y Meng H. Increased prolactin is an adaptive response to protect against metabolic disorders in obesity. Endocr Pract (2021) 27(7):728–35. doi: 10.1016/j.eprac.2021.01.002 33637446

[6] Liu J Zhang L Fu J Wang Q Wang G. Circulating prolactin level is increased in metabolically healthy obesity. Endocr Connect (2021) 10(4):484–91. doi: 10.1530/EC-21-0040 PMC8111314 33794504

[7] Larson BA Sinha YN Vanderlaan WP. Serum growth hormone and prolactin during and after the development of the obese-hyperglycemic syndrome in mice. Endocrinology (1976) 98(1):139–45. doi: 10.1210/endo-98-1-139 942910

[8] Sinha YN Thomas JW Salocks CB Wickes MA VanderLaan WP. Prolactin and growth hormone secretion in diet-induced obesity in mice. Horm Metab Res (1977) 9(4):277–82. doi: 10.1055/s-0028-1093552 892692

[9] Sinha YN Baxter SR Larson BA Vanderlaan WP. Levels of prolactin, growth hormone and insulin in genetically diabetic (db/db) mice. Proc Soc Exp Biol Med (1979) 161(1):78–81. doi: 10.3181/00379727-161-40494 441075

[10] Lemini M Ruiz-Herrera X Ledesma-Colunga MG Diaz-Lezama N De Los Rios EA Lopez-Barrera F. Prolactin anterior pituitary expression and circulating levels are reduced in obese and diabetic rats: Role of TGF-beta and TNF-alpha. Am J Physiol Regul Integr Comp Physiol (2015) 308(9):R792–9. doi: 10.1152/ajpregu.00327.2014 25715833

[11] Holstad M Sandler S. Prolactin protects against diabetes induced by multiple low doses of streptozotocin in mice. J Endocrinol (1999) 163(2):229–34. doi: 10.1677/joe.0.1630229 10556772

[12] Park S Kim DS Daily JW Kim SH. Serum prolactin concentrations determine whether they improve or impair beta-cell function and insulin sensitivity in diabetic rats. Diabetes Metab Res Rev (2011) 27(6):564–74. doi: 10.1002/dmrr.1215 21557442

[13] Ramirez-Hernandez G Adan-Castro E Diaz-Lezama N Ruiz-Herrera X Martinez de la Escalera G Macotela Y. Global deletion of the prolactin receptor aggravates streptozotocin-induced diabetes in mice. Front Endocrinol (Lausanne) (2021) 12:619696. doi: 10.3389/fendo.2021.619696 33746901 PMC7973366

[14] Yu J Xiao F Zhang Q Liu B Guo Y Lv Z. PRLR regulates hepatic insulin sensitivity in mice *via* STAT5. Diabetes (2013) 62(9):3103–13. doi: 10.2337/db13-0182 PMC3749345 23775766

[15] Augustine RA Ladyman SR Grattan DR. From feeding one to feeding many: hormone-induced changes in bodyweight homeostasis during pregnancy. J Physiol (2008) 586(2):387–97. doi: 10.1113/jphysiol.2007.146316 PMC2375600 18033810

[16] Zeng Z Liu F Li S. Metabolic adaptations in pregnancy: A review. Ann Nutr Metab (2017) 70(1):59–65. doi: 10.1159/000459633 28297696

[17] Sonagra AD Biradar SM KD Murthy DSJ. Normal pregnancy- a state of insulin resistance. J Clin Diagn Res (2014) 8(11):CC01–3. doi: 10.7860/JCDR/2014/10068.5081 PMC4290225 25584208

[18] Banerjee RR Cyphert HA Walker EM Chakravarthy H Peiris H Gu X. Gestational diabetes mellitus from inactivation of prolactin receptor and MafB in islet beta-cells. Diabetes (2016) 65(8):2331–41. doi: 10.2337/db15-1527 PMC4955982 27217483

[19] Huang C Snider F Cross JC. Prolactin receptor is required for normal glucose homeostasis and modulation of beta-cell mass during pregnancy. Endocrinology (2009) 150(4):1618–26. doi: 10.1210/en.2008-1003 19036882

[20] Nteeba J Kubota K Wang W Zhu H Vivian J Dai G. Pancreatic prolactin receptor signaling regulates maternal glucose homeostasis. J Endocrinol (2019) 241:71–83. doi: 10.1530/JOE-18-0518 PMC7189340 30798322

[21] Shrivastava V Lee M Lee D Pretorius M Radford B Makkar G. Beta cell adaptation to pregnancy requires prolactin action on both beta and non-beta cells. Sci Rep (2021) 11(1):10372. doi: 10.1038/s41598-021-89745-9 33990661 PMC8121891

[22] de Los Rios EA Ruiz-Herrera X Tinoco-Pantoja V Lopez-Barrera F Martinez de la Escalera G Clapp C. Impaired prolactin actions mediate altered offspring metabolism induced by maternal high-fat feeding during lactation. FASEB J (2018) 32(6):3457–70. doi: 10.1096/fj.201701154R 29401632

[23] Li J Rice MS Huang T Hankinson SE Clevenger CV Hu FB. Circulating prolactin concentrations and risk of type 2 diabetes in US women. Diabetologia (2018) 61(12):2549–60. doi: 10.1007/s00125-018-4733-9 PMC6309828 30306190

[24] Manshaei N Shakibaei F Fazilati M Salavati H Negahdary M Palizban A. An investigation of the association between the level of prolactin in serum and type II diabetes. Diabetes Metab Syndr (2019) 13(5):3035–41. doi: 10.1016/j.dsx.2018.07.007 30030156

[25] Wang T Xu Y Xu M Ning G Lu J Dai M. Circulating prolactin and risk of type 2 diabetes: A prospective study. Am J Epidemiol (2016) 184(4):295–301. doi: 10.1093/aje/kwv326 27466075

[26] Wang T Lu J Xu Y Li M Sun J Zhang J. Circulating prolactin associates with diabetes and impaired glucose regulation: A population-based study. Diabetes Care (2013) 36(7):1974–80. doi: 10.2337/dc12-1893 PMC3687322 23340889

[27] Balbach L Wallaschofski H Volzke H Nauck M Dorr M Haring R. Serum prolactin concentrations as risk factor of metabolic syndrome or type 2 diabetes? BMC Endocr Disord (2013) 13:12. doi: 10.1186/1472-6823-13-12 23517652 PMC3614874

[28] Chahar C Chahar K Ankit BS Gadhwal A Agrawal RP. Association of serum prolactin level with impaired glucose regulation and diabetes. J Assoc Physicians India (2017) 65(3):34–9. 28462541

[29] Retnakaran R Ye C Kramer CK Connelly PW Hanley AJ Sermer M. Maternal serum prolactin and prediction of postpartum beta-cell function and risk of Prediabetes/Diabetes. Diabetes Care (2016) 39(7):1250–8. doi: 10.2337/dc16-0043 27208323

[30] Zhang Z Piro AL Allalou A Alexeeff SE Dai FF Gunderson EP. Prolactin and maternal metabolism in women with a recent GDM pregnancy and links to future T2D: The SWIFT study. J Clin Endocrinol Metab (2022) 107:2652–65. doi: 10.1210/clinem/dgac346 PMC9387721 35666146

[31] Wagner R Heni M Linder K Ketterer C Peter A Bohm A. Age-dependent association of serum prolactin with glycaemia and insulin sensitivity in humans. Acta Diabetol (2014) 51(1):71–8. doi: 10.1007/s00592-013-0493-7 23836327

[32] Chirico V Cannavo S Lacquaniti A Salpietro V Mandolfino M Romeo PD. Prolactin in obese children: A bridge between inflammation and metabolic-endocrine dysfunction. Clin Endocrinol (Oxf) (2013) 79(4):537–44. doi: 10.1111/cen.12183 23445298

[33] Yang H Lin J Li H Liu Z Chen X Chen Q. Prolactin is associated with insulin resistance and beta-cell dysfunction in infertile women with polycystic ovary syndrome. Front Endocrinol (Lausanne) (2021) 12:571229. doi: 10.3389/fendo.2021.571229 33716958 PMC7947819

[34] Albu A Florea S Fica S. Is prolactin the missing link in adipose tissue dysfunction of polycystic ovary syndrome patients? Endocrine (2016) 51(1):163–73. doi: 10.1007/s12020-015-0655-1 26067083

[35] Kvasnickova H Hampl R Vondra K. DHEA, DHEAS and prolactin correlate with glucose control parameters in women of fertile age with type-1 diabetes mellitus. Physiol Res (2015) 64(Suppl 2):S255–8. doi: 10.33549/physiolres.933091 26680487

[36] Corona G Mannucci E Jannini EA Lotti F Ricca V Monami M. Hypoprolactinemia: A new clinical syndrome in patients with sexual dysfunction. J Sex Med (2009) 6(5):1457–66. doi: 10.1111/j.1743-6109.2008.01206.x 19210705

[37] Corona G Wu FC Rastrelli G Lee DM Forti G O'Connor DB. Low prolactin is associated with sexual dysfunction and psychological or metabolic disturbances in middle-aged and elderly men: The European Male aging study (EMAS). J Sex Med (2014) 11(1):240–53. doi: 10.1111/jsm.12327 24345293

[38] Glintborg D Altinok M Mumm H Buch K Ravn P Andersen M. Prolactin is associated with metabolic risk and cortisol in 1007 women with polycystic ovary syndrome. Hum Reprod (2014) 29(8):1773–9. doi: 10.1093/humrep/deu133 24903198

[39] Yang H Di J Pan J Yu R Teng Y Cai Z. The Association Between Prolactin and MetabolicParameters in PCOS Women: A Retrospective Analysis. Frontiers inEndocrinology (2020). doi: 10.3389/fendo.2020.00263 PMC7235367 32477263

[40] Corona G Rastrelli G Boddi V Monami M Melani C Balzi D. Prolactin levels independently predict major cardiovascular events in patients with erectile dysfunction. Int J Androl (2011) 34(3):217–24. doi: 10.1111/j.1365-2605.2010.01076.x 20522124

[41] Zhang P Ge Z Wang H Feng W Sun X Chu X. Prolactin improves hepatic steatosis *via* CD36 pathway. J Hepatol (2018) 68(6):1247–55. doi: 10.1016/j.jhep.2018.01.035 29452209

[42] Faria de Castro L Alves Dos Santos Á Augusto Casulari L Ansaneli Naves L Amorim Amato A. Association between variations of physiological prolactin serum levels and the risk of type 2 diabetes: A systematic review and meta-analysis. Diabetes Res Clin Pract (2020) 166:108247. doi: 10.1016/j.diabres.2020.108247 32505717

[43] Kahn CR Wang G Lee KY. Altered adipose tissue and adipocyte function in the pathogenesis of metabolic syndrome. J Clin Invest (2019) 129(10):3990–4000. doi: 10.1172/JCI129187 31573548 PMC6763230

[44] Favre G Legueult K Pradier C Raffaelli C Ichai C Iannelli A. Visceral fat is associated to the severity of COVID-19. Metabolism (2021) 115:154440. doi: 10.1016/j.metabol.2020.154440 33246009 PMC7685947

[45] McLaughlin T Lamendola C Liu A Abbasi F. Preferential fat deposition in subcutaneous versus visceral depots is associated with insulin sensitivity. J Clin Endocrinol Metab (2011) 96(11):E1756–E60. doi: 10.1210/jc.2011-0615 PMC3205890 21865361

[46] Lee CG Lee JK Kang YS Shin S Kim JH Lim YJ. Visceral abdominal obesity is associated with an increased risk of irritable bowel syndrome. Am J Gastroenterol (2015) 110(2):310–9. doi: 10.1038/ajg.2014.422 25583325

[47] Alberti KG Eckel RH Grundy SM Zimmet PZ Cleeman JI Donato KA. Harmonizing the metabolic syndrome: A joint interim statement of the international diabetes federation task force on epidemiology and prevention; national heart, lung, and blood institute; American heart association; world heart federation; international atherosclerosis society; and international association for the study of obesity. Circulation (2009) 120(16):1640–5. doi: 10.1161/CIRCULATIONAHA.109.192644 19805654

[48] Zhang P Feng W Chu X Sun X Zhu D Bi Y. A newly noninvasive model for prediction of non-alcoholic fatty liver disease: Utility of serum prolactin levels. BMC Gastroenterol (2019) 19(1):202. doi: 10.1186/s12876-019-1120-z 31775658 PMC6882057

[49] Phillipps HR Yip SH Grattan DR. Patterns of prolactin secretion. Mol Cell Endocrinol (2020) 502:110679. doi: 10.1016/j.mce.2019.110679 31843563

[50] Bauman DE Currie WB. Partitioning of nutrients during pregnancy and lactation: A review of mechanisms involving homeostasis and homeorhesis. J Dairy Sci (1980) 63(9):1514–29. doi: 10.3168/jds.S0022-0302(80)83111-0 7000867

[51] Ben-Jonathan N Hugo ER Brandebourg TD LaPensee CR. Focus on prolactin as a metabolic hormone. Trends Endocrinol Metab (2006) 17(3):110–6. doi: 10.1016/j.tem.2006.02.005 16517173

[52] Lopez-Vicchi F De Winne C Brie B Sorianello E Ladyman SR Becu-Villalobos D. Metabolic functions of prolactin: Physiological and pathological aspects. J Neuroendocrinol (2020) 32(11):e12888. doi: 10.1111/jne.12888 33463813

[53] Brelje TC Parsons JA Sorenson RL. Regulation of islet beta-cell proliferation by prolactin in rat islets. Diabetes (1994) 43(2):263–73. doi: 10.2337/diab.43.2.263 7904577

[54] Terra LF Garay-Malpartida MH Wailemann RA Sogayar MC Labriola L. Recombinant human prolactin promotes human beta cell survival *via* inhibition of extrinsic and intrinsic apoptosis pathways. Diabetologia (2011) 54(6):1388–97. doi: 10.1007/s00125-011-2102-z 21394492

[55] Auffret J Freemark M Carre N Mathieu Y Tourrel-Cuzin C Lombes M. Defective prolactin signaling impairs pancreatic beta-cell development during the perinatal period. Am J Physiol Endocrinol Metab (2013) 305(10):E1309–18. doi: 10.1152/ajpendo.00636.2012 PMC3840213 24064341

[56] Kim H Toyofuku Y Lynn FC Chak E Uchida T Mizukami H. Serotonin regulates pancreatic beta cell mass during pregnancy. Nat Med (2010) 16(7):804–8. doi: 10.1038/nm.2173 PMC2921604 20581837

[57] Kondegowda NG Fenutria R Pollack IR Orthofer M Garcia-Ocana A Penninger JM. Osteoprotegerin and denosumab stimulate human beta cell proliferation through inhibition of the receptor activator of NF-kappaB ligand pathway. Cell Metab (2015) 22(1):77–85. doi: 10.1016/j.cmet.2015.05.021 26094891 PMC4597781

[58] Xu Y Wang X Gao L Zhu J Zhang H Shi H. Prolactin-stimulated survivin induction is required for beta cell mass expansion during pregnancy in mice. Diabetologia (2015) 58(9):2064–73. doi: 10.1007/s00125-015-3670-0 26099856

[59] Weinhaus AJ Stout LE Sorenson RL. Glucokinase, hexokinase, glucose transporter 2, and glucose metabolism in islets during pregnancy and prolactin-treated islets *in vitro*: Mechanisms for long term up-regulation of islets. Endocrinology (1996) 137(5):1640–9. doi: 10.1210/endo.137.5.8612496 8612496

[60] Weinhaus AJ Stout LE Bhagroo NV Brelje TC Sorenson RL. Regulation of glucokinase in pancreatic islets by prolactin: A mechanism for increasing glucose-stimulated insulin secretion during pregnancy. J Endocrinol (2007) 193(3):367–81. doi: 10.1677/JOE-07-0043 17535875

[61] Moreno-Carranza B Goya-Arce M Vega C Adán N Triebel J López-Barrera F. Prolactin promotes normal liver growth, survival, and regeneration in rodents: effects on hepatic IL-6, suppressor of cytokine signaling-3, and angiogenesis. Am J Physiol Regul Integr Comp Physiol (2013) 305(7):R720–6. doi: 10.1152/ajpregu.00282.2013 23948778

[62] Shao S Yao Z Lu J Song Y He Z Yu C. Ablation of prolactin receptor increases hepatic triglyceride accumulation. Biochem Biophys Res Commun (2018) 498(3):693–9. doi: 10.1016/j.bbrc.2018.03.048 29524401

[63] Berlanga JJ Gualillo O Buteau H Applanat M Kelly PA Edery M. Prolactin activates tyrosyl phosphorylation of insulin receptor substrate 1 and phosphatidylinositol-3-OH kinase. J Biol Chem (1997) 272(4):2050–2. doi: 10.1074/jbc.272.4.2050 8999900

[64] Garcia-Martinez JM Calcabrini A Gonzalez L Martin-Forero E Agullo-Ortuno MT Simon V. A non-catalytic function of the src family tyrosine kinases controls prolactin-induced Jak2 signaling. Cell Signal (2010) 22(3):415–26. doi: 10.1016/j.cellsig.2009.10.013 19892015

[65] Brandebourg TD Bown JL Ben-Jonathan N. Prolactin upregulates its receptors and inhibits lipolysis and leptin release in male rat adipose tissue. Biochem Biophys Res Commun (2007) 357(2):408–13. doi: 10.1016/j.bbrc.2007.03.168 PMC1885988 17433256

[66] Ling C Svensson L Oden B Weijdegard B Eden B Eden S. Identification of functional prolactin (PRL) receptor gene expression: PRL inhibits lipoprotein lipase activity in human white adipose tissue. J Clin Endocrinol Metab (2003) 88(4):1804–8. doi: 10.1210/jc.2002-021137 12679477

[67] LaPensee CR Horseman ND Tso P Brandebourg TD Hugo ER Ben-Jonathan N. The prolactin-deficient mouse has an unaltered metabolic phenotype. Endocrinology (2006) 147(10):4638–45. doi: 10.1210/en.2006-0487 16809445

[68] Nanbu-Wakao R Fujitani Y Masuho Y Muramatu M Wakao H. Prolactin enhances CCAAT enhancer-binding protein-beta (C/EBP beta) and peroxisome proliferator-activated receptor gamma (PPAR gamma) messenger RNA expression and stimulates adipogenic conversion of NIH-3T3 cells. Mol Endocrinol (2000) 14(2):307–16.doi: 10.1210/mend.14.2.0420 10674402

[69] Stewart WC Baugh JE Jr. Floyd ZE Stephens JM. STAT 5 activators can replace the requirement of FBS in the adipogenesis of 3T3-L1 cells. Biochem Biophys Res Commun (2004) 324(1):355–9. doi: 10.1016/j.bbrc.2004.09.053 15465026

[70] Viengchareun S Servel N Feve B Freemark M Lombes M Binart N. Prolactin receptor signaling is essential for perinatal brown adipocyte function: A role for insulin-like growth factor-2. PLoS One (2008) 3(2):e1535. doi: 10.1371/journal.pone.0001535 18253483 PMC2212135

[71] Zinger M McFarland M Ben-Jonathan N. Prolactin expression and secretion by human breast glandular and adipose tissue explants. J Clin Endocrinol Metab (2003) 88(2):689–96. doi: 10.1210/jc.2002-021255 12574200

[72] Freemark M Fleenor D Driscoll P Binart N Kelly P. Body weight and fat deposition in prolactin receptor-deficient mice. Endocrinology (2001) 142(2):532–7. doi: 10.1210/endo.142.2.7979 11159821

[73] Flint DJ Binart N Boumard S Kopchick JJ Kelly P. Developmental aspects of adipose tissue in GH receptor and prolactin receptor gene disrupted mice: site-specific effects upon proliferation, differentiation and hormone sensitivity. J Endocrinol (2006) 191(1):101–11. doi: 10.1677/joe.1.06939 17065393

[74] Auffret J Viengchareun S Carre N Denis RG Magnan C Marie PY. Beige differentiation of adipose depots in mice lacking prolactin receptor protects against high-fat-diet-induced obesity. FASEB J (2012) 26(9):3728–37. doi: 10.1096/fj.12-204958 22637534

[75] Xiao F Xia T Lv Z Zhang Q Xiao Y Yu J. Central prolactin receptors (PRLRs) regulate hepatic insulin sensitivity in mice *via* signal transducer and activator of transcription 5 (STAT5) and the vagus nerve. Diabetologia (2014) 57(10):2136–44. doi: 10.1007/s00125-014-3336-3 25064125

[76] Park S Kang S Lee HW Ko BS. Central prolactin modulates insulin sensitivity and insulin secretion in diabetic rats. Neuroendocrinology (2012) 95(4):332–43. doi: 10.1159/000336501 22441304

[77] Peuskens J Pani L Detraux J De Hert M. The effects of novel and newly approved antipsychotics on serum prolactin levels: A comprehensive review. CNS Drugs (2014) 28(5):421–53. doi: 10.1007/s40263-014-0157-3 PMC4022988 24677189

[78] Torre DL Falorni A. Pharmacological causes of hyperprolactinemia. Ther Clin Risk Manage (2007) 3(5):929–51. PMC2376090 18473017

[79] Pillinger T McCutcheon RA Vano L Mizuno Y Arumuham A Hindley G. Comparative effects of 18 antipsychotics on metabolic function in patients with schizophrenia, predictors of metabolic dysregulation, and association with psychopathology: A systematic review and network meta-analysis. Lancet Psychiatry (2020) 7(1):64–77. doi: 10.1016/S2215-0366(19)30416-X 31860457 PMC7029416

[80] Zhang Y Wang Q Reynolds GP Yue W Deng W Yan H. Metabolic effects of 7 antipsychotics on patients with schizophrenia: A short-term, randomized, open-label, multicenter, pharmacologic trial. J Clin Psychiatry (2020) 81(3):19m12785. doi: 10.4088/JCP.19m12785 32237292

[81] Henderson DC. Managing weight gain and metabolic issues in patients treated with atypical antipsychotics. J Clin Psychiatry (2008) 69(2):e04. doi: 10.4088/JCP.0208e04 18363448

[82] Roix JJ DeCrescenzo GA Cheung PH Ciallella JR Sulpice T Saha S. Effect of the antipsychotic agent amisulpride on glucose lowering and insulin secretion. Diabetes Obes Metab (2012) 14(4):329–34. doi: 10.1111/j.1463-1326.2011.01529.x 22059694

[83] McKeage K Plosker GL. Amisulpride: A review of its use in the management of schizophrenia. CNS Drugs (2004) 18(13):933–56. doi: 10.2165/00023210-200418130-00007 15521794

[84] Robles-Osorio ML García-Franco R Núñez-Amaro CD Mira-Lorenzo X Ramírez-Neria P Hernández W. Basis and design of a randomized clinical trial to evaluate the effect of levosulpiride on retinal alterations in patients with diabetic retinopathy and diabetic macular edema. Front Endocrinol (Lausanne) (2018) 9:242. doi: 10.3389/fendo.2018.00242 29896154 PMC5986911

[85] Defronzo RA. Bromocriptine: a sympatholytic, d2-dopamine agonist for the treatment of type 2 diabetes. Diabetes Care (2011) 34(4):789–94. doi: 10.2337/dc11-0064 PMC3064029 21447659

[86] Framnes-DeBoer SN Bakke E Yalamanchili S Peterson H Sandoval DA Seeley RJ. Bromocriptine improves glucose tolerance independent of circadian timing, prolactin, or the melanocortin-4 receptor. Am J Physiol Endocrinol Metab (2020) 318(1):E62–71. doi: 10.1152/ajpendo.00325.2019 PMC6985791 31794265

